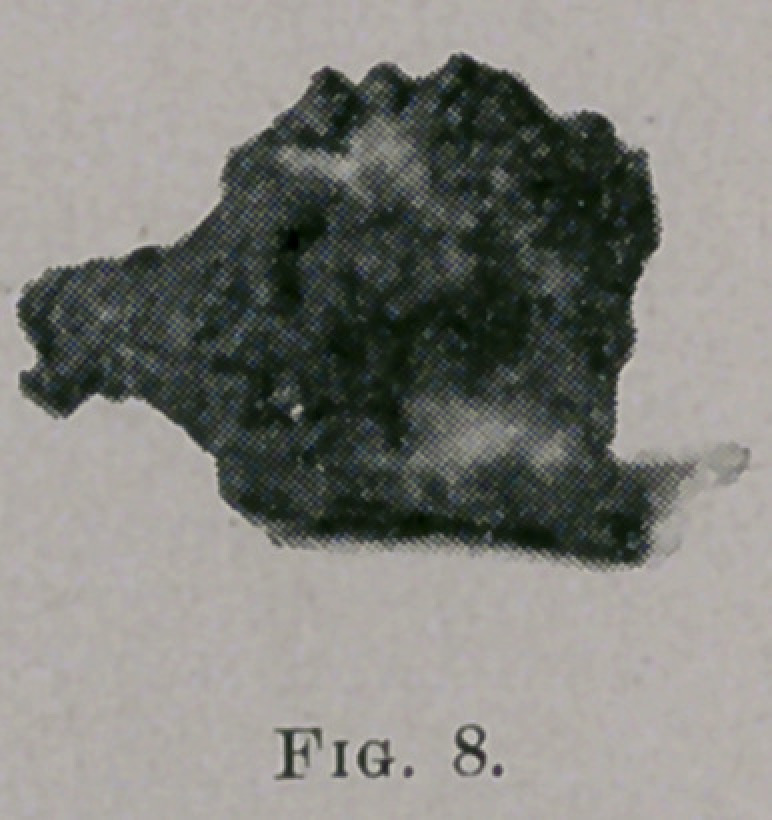# Nasal Stenosis1Read before the Steuben County Medical Society.

**Published:** 1895-05

**Authors:** S. Mitchell

**Affiliations:** Hornellsville, N. Y.; Oculist and Aurist to St. James’s Mercy Hospital; Oculist to the N. Y., L. E. & W. R. R.


Buffalo Medical and Surgical Journal

Vol. XXXIV. MAY, 1895. No. 10.

### Original Communications.

### NASAL STENOSIS.[1]

By S. MITCHELL, M. D., Hornellsville, N. Y.

Oculist and Aurist to St. James's Mercy Hospital ; Oculist to the N. Y., L. E. & W. R. R.

Nasal stenosis is an affection that the busy practitioner is almost daily called upon to treat and yet one that yields very unsatisfactory results, as ordinarily treated. Most of these cases come for treatment with the diagnosis of "catarrh" already made for us and with the history of having used Nely's Cream Balm, Page's Catarrh Remedy, Hayne's Monarch Catarrh Cure and a dozen other remedies that are so eloquently advertised in all the papers, with the standing offer of $500 or $1,000 for a case that their remedy will not cure. Now and then a case comes that has not used any of these patent nostrums, as did an old Irish woman, who applied to me a short time since for treatment of her catarrh, stating, "I have niver used onything for't but a little salt and wather sniffed up me nose to wash me brains out." Too often it happens that these ready-made diagnoses of catarrh are accepted by many of us who pride ourselves upon our shrewdness in making a diagnosis and, falling in with the generally accepted idea that catarrh cannot be cured, we do not trouble ourselves to search for any better remedy than the old Irish woman's brain irrigating fluid. When the one symptom—namely, that of obstructed nasal respiration, has existed for a long time, it should lead us to make a thorough examination before sending our patient away with simply a douche or a spray. In a majority of such cases we will find that a little surgery, such as I shall have occasion to speak of when I come to that part of my subject, will often do more to cure our patient than years of treatment by the use of applications, sprays and douches. The armamentarium necessary for diagnosis need not

---

1. Read before the Steuben County Medical Society.

be so very extensive. A head mirror, with a focal distance of eight to twelve inches, a nasal speculum, two or three throat mirrors and a tongue depressor. Then, with the aid of a good lamp, a pretty satisfactory diagnosis can be made, and treatment more satisfactorily and scientifically applied.

In speaking of the etiology or cause of nasal stenosis, the pathological conditions found in the same will necessarily be included.

### ETIOLOGY.

The causes that lead to stenosis of one or both nasal cavities may be congenital or acquired. The most common congenital condition producing stenosis is deviation of the septum, either of its cartilaginous or bony portion or of both. This condition very rarely causes stenosis of but one nostril, there being a corresponding increase of the caliber of the other, which increase is often compensated for as age advances by a hypertrophy of the middle turbinated bone. Deviation of the septum is not always a congenital condition, as will be shown further on. Bony projections from and malformations of the vomer, ethmoid and turbinated bones frequently give rise to partial or complete stenosis. Malformation and displacement of the cartilaginous portion of the septum are also among the congenital conditions. Among the acquired conditions also occurs deviation of the septum, brought about by blows upon the nose or by pressure brought to bear upon it from directly in front, from above or from either side. My experience has taught me that the great majority of deviations take place toward the right side, thus causing stenosis of the right nostril. Also, that they occur more frequently with men than women. I am unable to explain why the septum should be more frequently deviated to the right than to the left, but its frequent occurrence in men is readily explained by the fact that they are more exposed to blows and injuries about the face than women.

Next come exostoses or morbid bony growths from the perpendicular plate of the ethmoid, from the vomer and turbinated bones.

These growths occur more frequently from the septum, and rarely reach sufficient size to alone occlude the nostril upon which they encroach, but they are often met by an hypertrophied turbinated bone, and between the two the stenosis is complete.

Ecchondromata, or morbid growths from the cartilaginous portion of the septum, occur with about equal frequency, as do exostoses ; and as they generally come at the anterior nares, where the nostrils are easily occluded, they complete the stenosis without the help of an hypertrophied turbinated bone. Recently I had occasion to operate upon a case of this sort, where the morbid growth from the cartilage had reached the size of a small chestnut, completely closing the right nostril. This growth was removed by means of the nasal saw, giving as free respiration through this nostril as the other enjoyed. The operation was rendered completely painless by injecting five minims of a 10 per cent. solution of cocaine into the mucous membrane covering the growth. These cartilaginous growths sometimes form a ridge parallel with the base of the septum, where they are equally troublesome, as in this position they frequently come in conjunction with the inferior turbinated bone, thus making a complete bridge, which might be mistaken for the floor of the nasal cavity.

Now comes hypertrophy of the epithelium and sub-mucous layer and chronic distension of the corpora cavenosa that cover the turbinated bones or chronic hypertrophic rhinitis. This is perhaps the most common cause of nasal stenosis we meet with in this climate. Coming on as a result of frequent attacks of acute coryza or cold in the head, it gives the peculiar nasal twang to the voice so common among the dwellers north of Mason and Dixon's line that marks us as "Yankees" the world over. The mucous membrane covering the turbinated bones is very richly supplied with blood by large venous sinuses, composing the corpora cavenosa, lying beneath the epithelium and sub-mucous layers. During an attack of acute rhinitis, these sinuses become engorged with blood, causing a temporary increase in the thickness of the mucous membrane. If these engorgements are often repeated, there is a stasis of blood in the sinuses, serous exudation takes place, new connective tissue is formed and a consequent permanent increase of the thickness of the mucous membrane. Figs. 2 and 3 illustrate the changes, above described, very nicely. Fig. 1 shows the membrane in a natural and healthy condition. Fig. 2 shows the change that takes place during an attack of acute rhinitis, where there is great distension of the venous sinuses. Fig. 3 gives a good idea of the condition of the membrane in chronic rhinitis, where there has been an exudation of plastic material and hypertrophy of the mucous tissues resulting. These plates are copied from Sajous' work on Diseases of the Nose and Throat.

The next frequent cause of stenosis is hypertrophy of the middle and inferior turbinated bones. This condition is frequently

FIG. 1.

FIG. 2.

FIG. 3.

FIG. 4.

FIG. 5.

FIG. 6.

FIG. 7.

FIG. 8.

MITCHELL—NASAL STENOSIS.

associated with the morbid state of the mucous tissues covering them, as just described. These hypertrophies may be of the anterior or posterior part of the bone only or of the whole structure. The posterior hypertrophy presents two appearances upon rhinoscopic examination. One, a dark red, almost purple color, presenting a granular appearance not unlike a large raspberry. Fig. 4 is a photograph of one that I removed with the cold wire snare recently from the inferior turbinated bone of a young man, a painter by occupation. The other a pearly white. They are sometimes so large as to completely close the posterior nares and extend into the naso-pharynx, causing deafness, as will be shown further on. I recently removed, by means of the cold wire snare, a portion of an hypertrophied inferior turbinated bone from a young man about thirty years of age, who came to me complaining of deafness in the left ear. I could find no other cause for the deafness than this enlarged bone, which, I inferred, was interfering with the free passage of air to the tympanic cavity, either from closure of the Eustachian orifice by direct pressure, or a complete stasis of the air as a result of the stenosis of this nasal passage. The hearing distance in this ear, as tested by the watch, was 30–60, and after the removal of this hypertrophy it quickly rose to 60–60 and so remained the last time the patient was seen. Fig. 5 shows the part of the bone that was removed in this case.

Tumors, both benign and malignant, frequently occur in the nasal cavities, causing more or less stenosis, according to their size and position. Sarcomatous and scirrhous tumors very rarely occur here. The most common are mucous and fibrous polypi, springing from the under surface of the overhanging turbinated bones, sometimes from the septum and not unfrequently from the vault of the pharynx. The presence of polypi are generally easy to ascertain, unless when by their increased growth they have pushed the overhanging turbinated bones over against the septum and nothing but this bone can be discovered upon examination. This occurred with me recently. A 10 per cent. solution of cocaine was applied to an hypertrophied inferior turbinated bone preparatory to using the cautery upon it; but by the constringing effect of the cocaine, the soft tissues were so reduced that I was able to discover several polypi and remove them. One of the most notable cases I have met with of polypoid growth was that of a young lady eighteen years of age, whose right nostril had been occluded for eight years by polypi. They could be easily seen at the anterior nares and a portion of a large one was visible projecting below the velum. A large number, about the size of chestnuts, were removed from the anterior nares at different sittings by means of the Jarvis snare. The large one in the posterior nares was removed in the following manner: a small gum catheter, carrying a strong cord, was passed through the nostril into the throat and the cord brought out through the mouth. By means of this cord a large loop of number five piano wire, previously threaded into a Jarvis snare, was drawn through the nostril and into the throat. Then, by passing the finger up behind the velum, the loop was adjusted about the attachments of the tumor and with a few turns of the milled nut the tumor dropped into the throat and was removed from the mouth. This tumor is shown in its natural size in Fig. 6.

These tumors present almost every conceivable shape and size. They are often molded into the many peculiar forms that they present by the shape of that part of the cavity which they have inhabited. Fig. 7 is a photograph of a tumor of this nature, natural size, that owes its peculiar shape to the place of its attachment. It was removed a few months ago from the naso-pharynx of a young lady who, by the way, had suffered for some time previous to its removal with asthma. It was attached by a very small pedicle to the mucous membrane in the region of the *fossa of Rosenmuller.* The removal was quite easily accomplished by means of a Bosworth snare, using a curved canula passed back of the velum from the mouth.

Adenoid vegetations sometimes form in the vault of the pharynx as a result of naso-pharyngeal catarrh. Their presence always affects the voice, causing the sufferer to speak thick, and if existing to any great extent will cause stenosis of one or both nasal cavities. This morbid condition occurs most frequently in children. Children thus affected are mouth breathers and with the half-open mouth and vacant countenance that such sufferers invariably present, they readily acquire the titles "blockheads," "stupid," "half-witted," and the like, titles which they quickly drop as soon as the vault of the pharynx is cleared of the adenoid vegetation.

Foreign bodies frequently find their way into the nasal cavity and by their presence and the swelling they occasion, cause stenosis of the same. If not removed they sometimes form the nucleus of calcareous concretions or rhinoliths. In Fig. 8 is shown a photograph, in its natural size, of a rhinolith that I removed about five years ago from the right nasal cavity of a gentleman forty-five years of age. There was complete occlusion of this nasal cavity from the presence of a large quantity of adenoid tissue with which the rhinolith was surrounded. He gave a history of having suffered from occlusion of the nasal passage for twenty-five years.

### SYMPTOMS.

The most prominent objective symptom of nasal stenosis will be obstructed nasal respiration. The subjects are mouth breathers. Another will be the peculiar tone of voice, with which we are all familiar. Loss of the sense of smell is not always a result, but frequently occurs. Pain is often present both in the nasal cavities over the frontal sinuses and through the temples, generally dull and heavy, but occasionally sharp and neuralgic in character. Asthma is also a frequent symptom, more often when the stenosis is caused by large hypertrophy of the posterior ends of the middle turbinated bones, or when the posterior nares and vault of pharynx is filled with adenoid vegetations or numerous small polypi.

I have met with two cases recently where there was stenosis of one nostril, as a result of an hypertrophy of the middle turbinated bones and causing in both quite severe neuralgia over the superior maxillary region of the same side. Deafness is a frequent accompaniment, due often, no doubt, to a static condition of the air in the naso-pharynx and a consequent collapse of the Eustachian tube; due frequently to a catarrhal condition of the tubes and tympanic cavities, aggravated by the stenosis. A mechanical closure of the Eustachian tubes may take place by an hypertrophy of the posterior ends of the middle or inferior turbinated bone, as no doubt was the condition in the case previously referred to, or by polypi, as was the case with the young lady, from whom the large polypus (see Fig. 6) was removed. Her first remark after its removal being, "Why, I can hear with this ear now," indicating the ear corresponding to the side from which the growth was removed. Another quite constant symptom is dulness of the intellect and inability to think clearly and consecutively upon any subject. Depression of spirits, morbid fears and aversion to society are often complained of. So, too, are hay fever or attacks of sneezing with coryza and lachrymation. These symptoms do not differ from simple periodical hyperesthetic rhinitis, except in that patients with permanent stenosis are rarely free from them, which symptoms are greatly aggravated during the summer months or hay fever season. Coughing frequently accompanies stenosis, caused by the secretions from the nasal cavity passing into the naso-pharynx and so finding its way into the larynx and acting as a constant source of irritation. The ocular symptoms are many and varied ; increased lachrymation, stricture of the nasal duct, from extension of the catarrhal inflammation from the lining membrane of the nose ; inflammation and swelling of the lachrymal sac or chronic dacrye-cystitis ; affections of the lachrymal sac and nasal duct, conjunctivitis, ulcers of the cornea, are some of the more common ocular troubles resulting directly and indirectly from nasal stenosis. Converging strabismus, temporary myopia and astigmatism may be caused by the irritation of nasal stenosis. In fact, I hardly ever treat any affection of the eyes without examining the nasal cavities and, when it is possible, remove whatever may be a source of irritation there.

### PROGNOSIS.

The prognosis will, of course, be largely influenced by the cause, but is universally favorable in stenosis from all variety of causes, provided the patient has sufficient pluck to pursue the treatment.

### TREATMENT.

In speaking of treatment I shall first mention the treatment of the most common form of stenosis—namely, that caused by hypertrophy of the mucous covering of the turbinated bones, chronic hypertrophic rhinitis. The chief aim here is to remove or reduce the thickened tissue. This may be done by both medicinal and surgical means. Where there is no hypertrophy of the bone associated with the thickened tissues, escharotics and astringents are of service. Among the escharotics, chromic and glacial acetic acid are perhaps about the best. These can be applied by means of an aluminum probe, slightly flattened and bent to be more convenient. (Before applying any of these, the parts should be sprayed with a 2 per cent. solution of cocaine.) The flattened portion (which should be about two inches long) is covered with absorbent cotton. The side that will be in contact with the septum is smeared with vaseline ; the other side carries the escharotic. The probe is then passed well back into the nasal cavity and the side that is covered with the escharotic is quickly applied to the thickened surface of the turbinated bone. These applications should be followed immediately by an alkaline spray to neutralize any excess of the acid. The applications may be made once or twice a week, until the cicatricial contraction following their use is sufficient to relieve the stenosis. The galvano-cautery is so handy and so much more thorough in its action that I seldom employ any other means in these cases. An 8 per cent. solution of cocaine applied by means of pledgets of cotton, not only produces anesthesia of the parts where the cautery is to be applied, but it also produces a temporary patency of the nasal cavity by emptying the venous sinuses of the greater part of the blood and makes the application of cautery much more convenient. The flat cautery knife is preferable. It is introduced and applied to the posterior part of the turbinated bone while cold, and as the current is turned on, the knife is drawn forward, cauterizing the whole surface of hypertrophied tissue, care being taken not to allow the knife to touch the septum, as this would favor an adhesion of the turbinated bone to the septum. If the knife is heated to a bright red heat and cocaine thoroughly applied, there is but little pain from these applications. The cautery may be used in most cases twice a week; the slough usually comes away at the end of three days. During the treatment, patients should be directed to use a small hand atomizer, with which the nasal cavities can be well sprayed and cleaned, using an alkaline fluid containing some antiseptic. The following is a modification of Dobel's solution that I usually prescribe:

℞ Acid carbolic .................................. gtt. x.
Sodii bicarb ..........................................
Sodii biborati .................................. aa gr. xv.
Pine needle extract ...................................
Listerine ......................................... aa ʒij.
Glycerini ..............................................
Aqua rosæ ........................................ aa ℥ss.
Aqua pura .................................. ad. q. s. ℥iv.

M. S.—Cleansing spray solution.

I do not place much dependence on astringent sprays and applications alone, nor do I often use bougies for gradual dilatation, as their use is tedious and painful. In many of these cases it is well to first remove a part of the thickened tissue by means of the snare, bistory or Knight's nasal scissors previous to applying the cautery, as it shortens the period of treatment. During the process of healing and for a time afterwards I usually direct my patients to use the following:

℞ Menthol ...............................................
Camphor ......................................... aa gr. x.
Eucalyptol ....................................... gtt. x.
Oil gaultheria .................................. gtt. ij.
Liquid albolene ............................. ad. q. s. ℥ij.

M. S.—Use in an albolene atomizer.

This is used immediately after using the cleansing spray by means of a special albolene atomizer, that can now be procured at any drug store.

Where there is bony hypertrophy, the galvano-cautery snare, the cold wire snare or the saw can be used. I prefer the cold wire snare and have never met with a case where its use was followed by severe hemorrhage. With the cautery snare there is not much danger from hemorrhage, but it is not as convenient to apply. After these operations and bleeding has ceased, insufflation of iodoform or aristol as an antiseptic precaution is necessary.

In the treatment of stenosis caused by deviation of the septum, it is always best to endeavor, if there is any possible chance of doing so, to open the nostril by removing a portion of the middle turbinated bone nearest the greatest convexity of the septum. If this is not possible, the septum should be straightened. When the deviation is only of the cartilaginous portion, this may be done by making a vertical incision through it, then with the finger introduced into the nostril encroached upon, push the septum over and retain it in place by using steel hair-lip pins suitably adjusted, or by means of plugs of carbolized okum. If plugs are used they must be removed at the end of twenty-four hours and the parts thoroughly sprayed with an antiseptic solution, permanganate of potash, one grain to the ounce, being as good as any. The pins may be left in for ten days or two weeks. Where there is deviation of the bony septum, the septal punch will come in play, choosing the die that best suits the case, to weaken the septum. After use the die is removed and the flat blades of the punch can be used to force the septum over, where it is kept in place by okum plugs.

Exostosis and ecchondromata are frequently associated with deviations of the septum. These, of course, must first be removed before any operation to straighten the septum is undertaken. Exostoses are removed with the nasal saw or by means of burrs and trephines. The operation is painful, as the cocaine, even if injected into the mucous membrane covering them, does not all affect the bone, but with a fine, sharp saw the operation is quickly done. Ecchondromata can often be removed with a strong bistory, but if they are of any considerable size the saw is preferable. A few drops of an 8 per cent. solution of cocaine injected into the mucous membrane covering these growths renders their removal perfectly painless.

In removing tumors the galvano-cautery and cold wire snare are the main reliance. In removing mucous polypi, I use the cold wire snare in all cases where it is possible to encircle them, as its use is much less painful than the forceps and not as liable to bring away a piece of the bone with the pedicle. The galvano-cautery snare is the best and safest instrument to use in the removal of fibrous polypi and tumors other than polypoid growths that have become perfectly organized and firmly and deeply attached. Small and sessile mucous polypi may be destroyed by the galvano-cautery puncture or by the application of escharotics. After removal of polypi, the place of their attachments must be thoroughly cauterized to prevent their return.

Adenoid vegetations in the vault of the pharynx can frequently be removed by the sharp finger nail of the forefinger, introduced behind the velum and swept across the vault. We can frequently in this way make the diagnosis of their presence and remove quite a quantity of the adenoid tissue at the same time. The curette, with a flexible shaft, in order that it may be bent, causing the blade to sweep in any direction, will be useful in removing tissue that cannot be reached by the finger nail. Cohen's post-nasal cutting forceps is probably the best instrument for thoroughly removing adenoid vegetations from the vault of the pharynx. The cold wire and galvano-cautery snares are frequently used.

Foreign bodies and rhinoliths can generally be removed with light but strong nasal forceps having the nasal cavity well illuminated. If from any cause forceps cannot be used, a strong probe, slightly flattened and bent at one end, can be gently passed well by the foreign body, either above or below it, and then by raising or lowering the free end of the probe, the intruder will be expelled.

In the foregoing paper I have attempted to present to you, in regular form, some of the more prominent features of an abnormal condition, rather than a disease *per se*, but brought about by a great variety of pathological changes. The subject is a large one and I have only been able to touch upon the more important points, and that too, I fear, at the risk of becoming an infliction upon a good-natured society.

---

New Treatment for Hydrocele.—Neuman (*St. Louis Med. and Surg. Jour.*) simply opens the hydrocele and leaves the canula in for two or three days, taking pains to surround it with antiseptic wool. The irritation of the canula is sufficient for drainage, without causing inflammation.

## Figures and Tables

**Fig. 1. f1:**
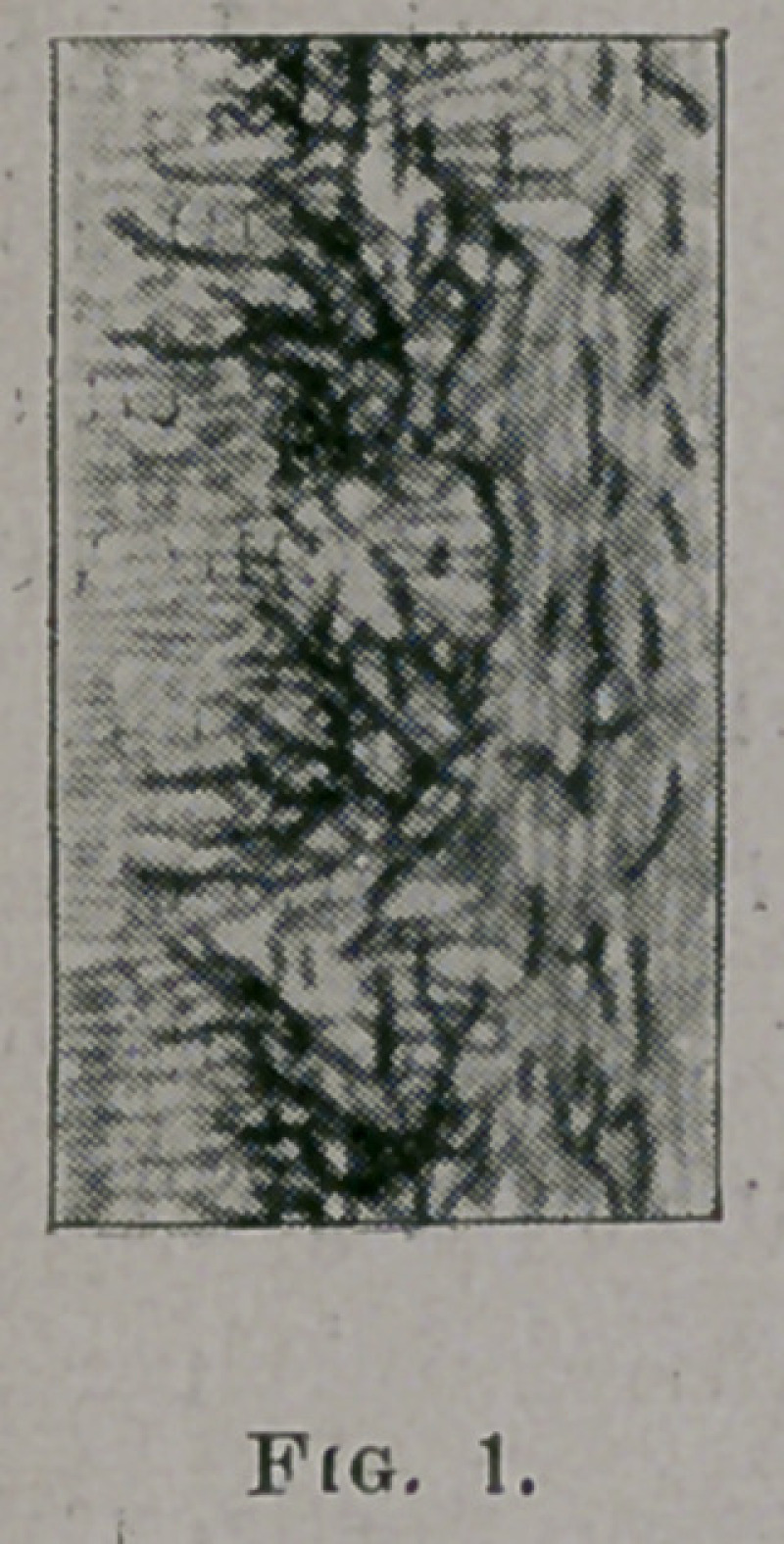


**Fig. 2. f2:**
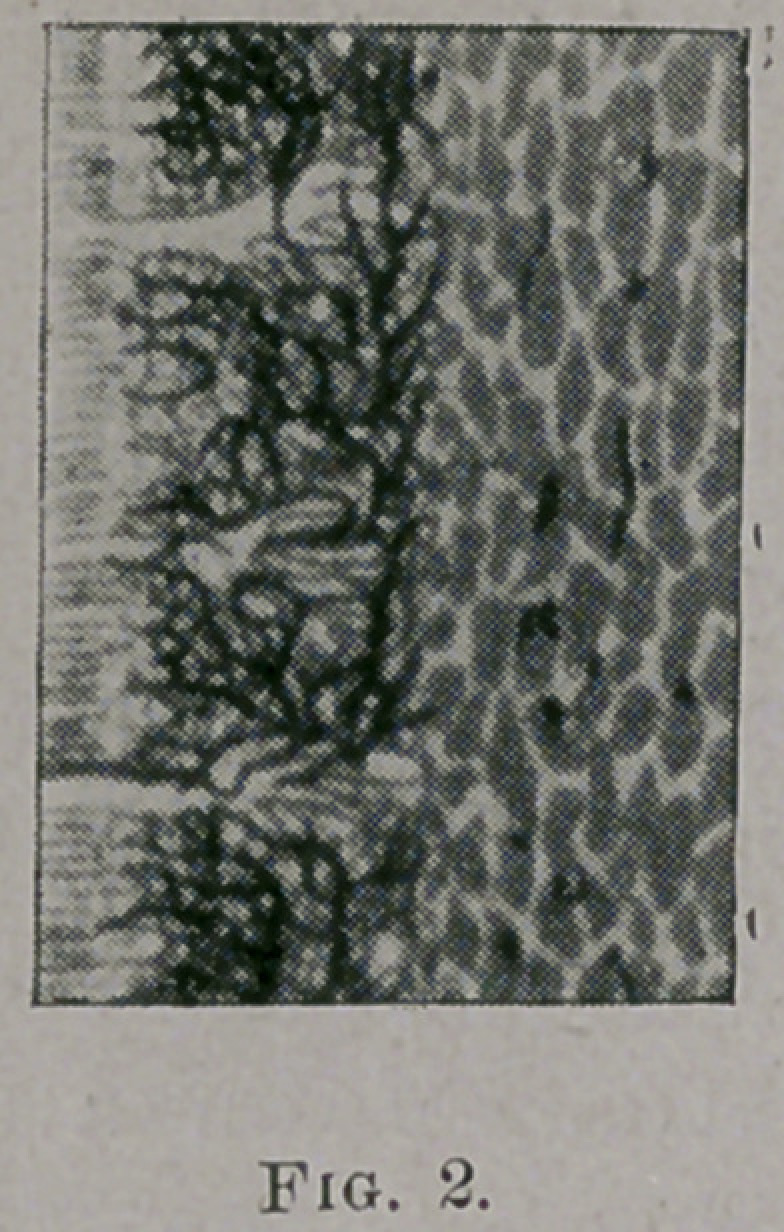


**Fig. 3. f3:**
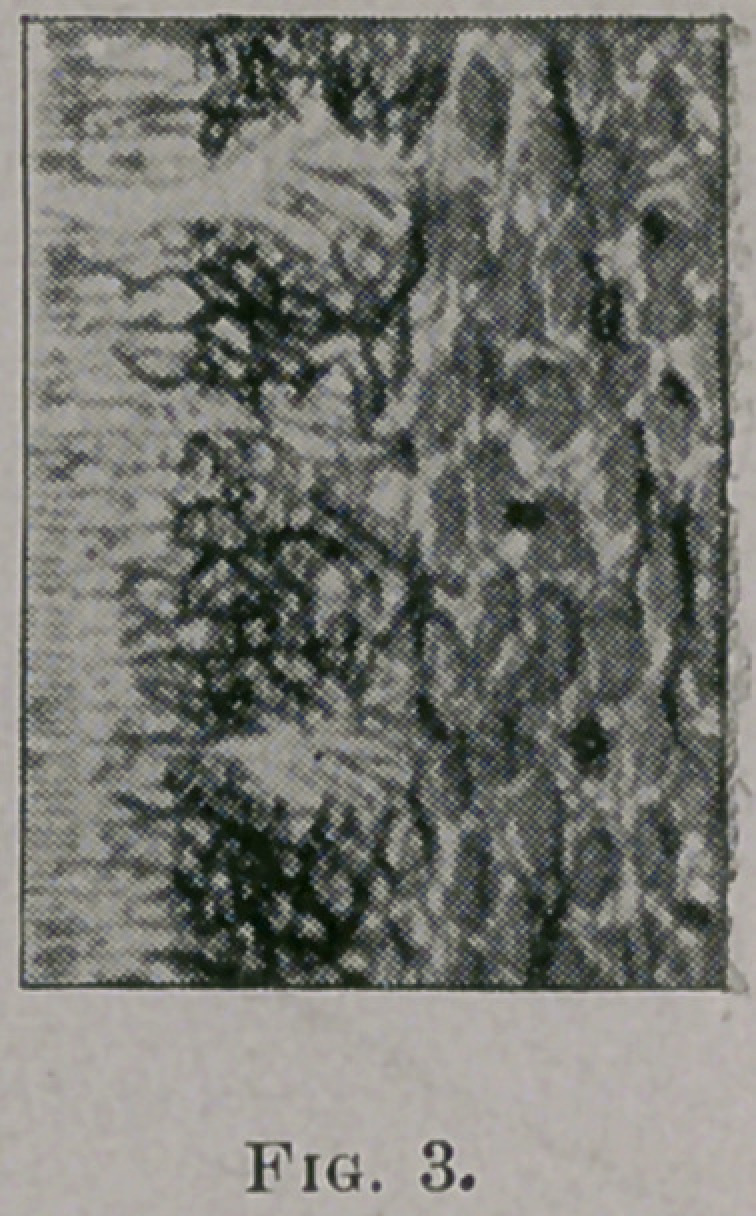


**Fig. 4. f4:**
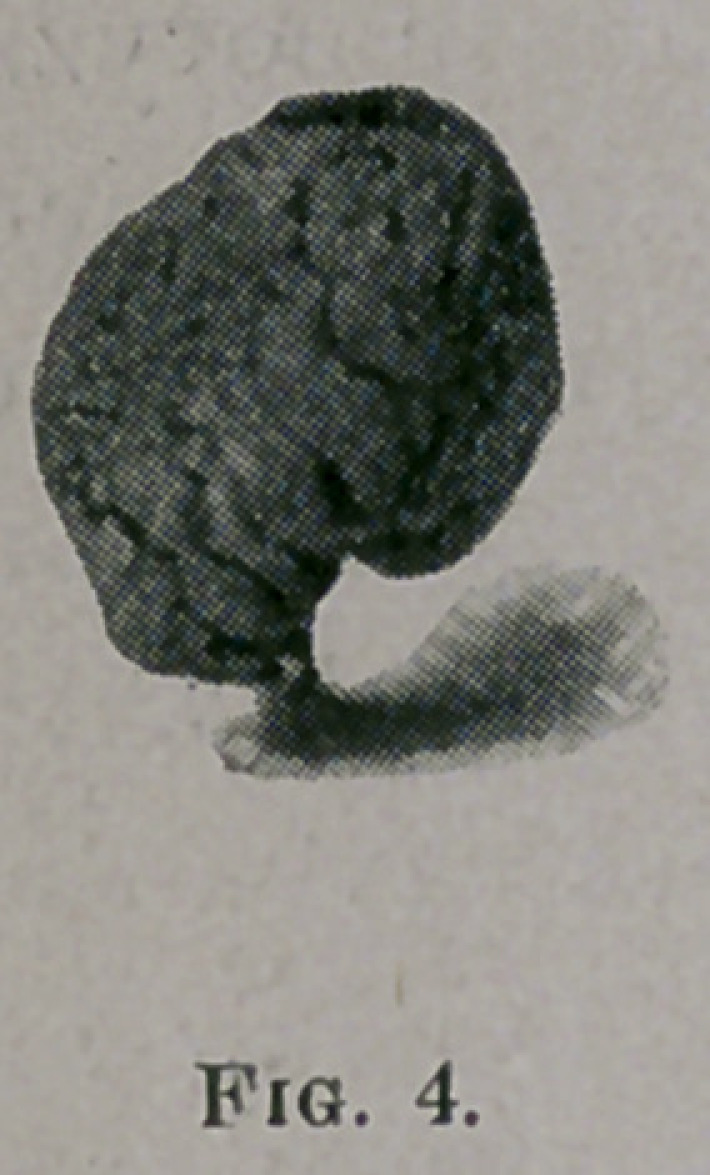


**Fig. 5. f5:**
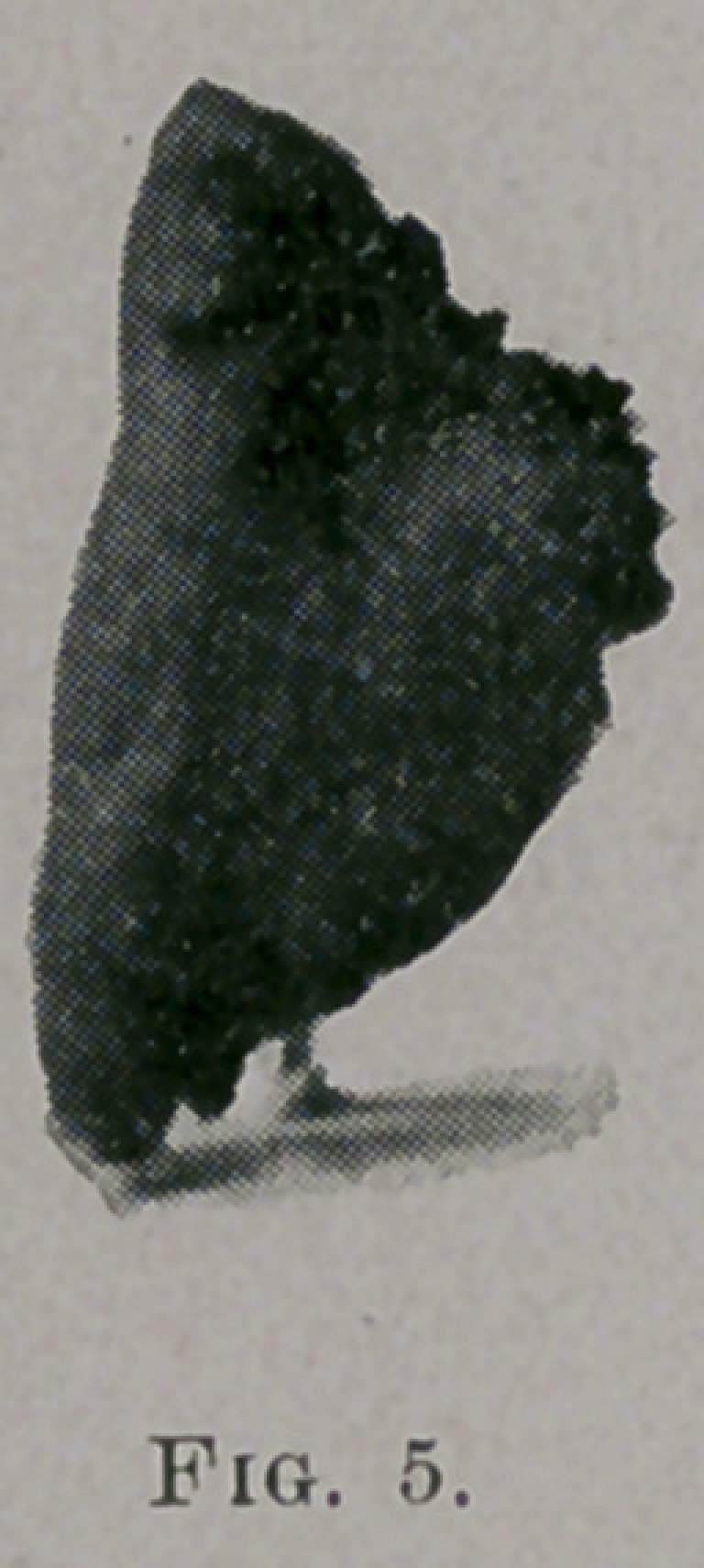


**Fig. 6. f6:**
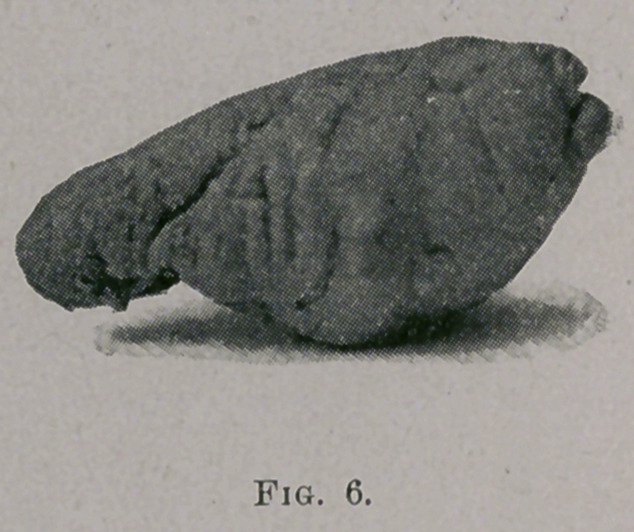


**Fig. 7. f7:**
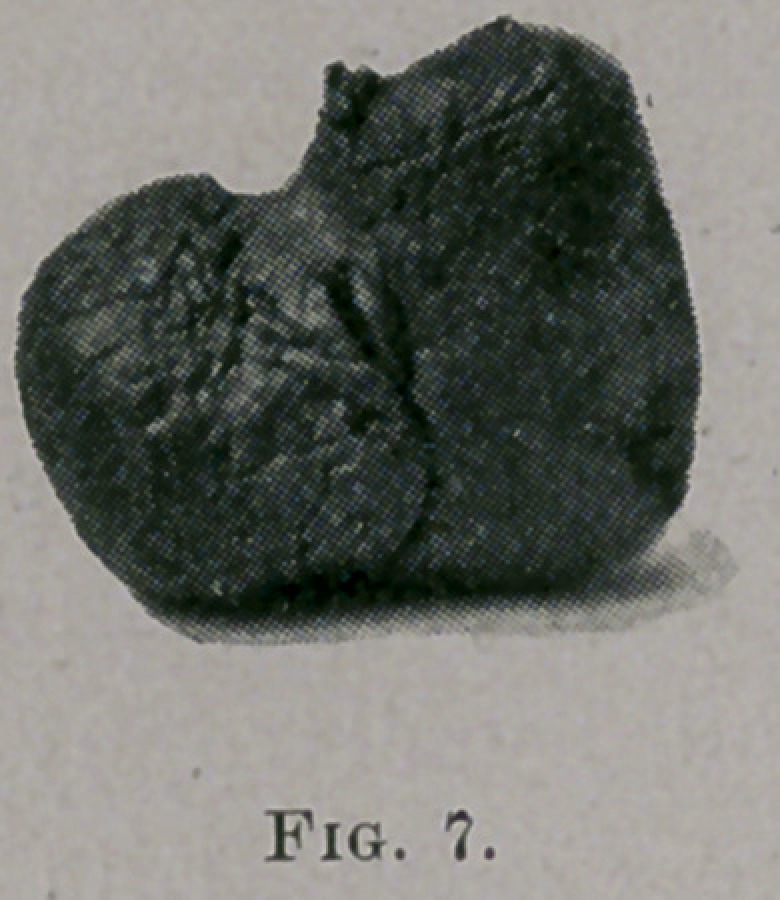


**Fig. 8. f8:**